# The role of the implementation of policies for the prevention of exposure to Radon in Brazil—a strategy for controlling the risk of developing lung cancer

**DOI:** 10.3332/ecancer.2015.572

**Published:** 2015-09-14

**Authors:** Aline da Rocha Lino, Carina Meira Abrahão, Marcus Paulo Fernandes Amarante, Marcelo Rocha de Sousa Cruz

**Affiliations:** Rua Martiniano de Carvalho, 965, Bela Vista, São Paul-SP, Brazil, CEP 01321-001

**Keywords:** cancer, radon, lung

## Abstract

Lung cancer is the leading cause of cancer death in the United States and other industrialised countries. The most important risk factor is active smoking. However, given the increased incidence of lung cancer in non-smokers, it is necessary to improve knowledge regarding other risk factors. Radon (Rn) is a noble gas and is the most important natural source of human exposure to ionizing radiation. Exposure to high levels of this radioactive gas is related to an increased risk of developing lung cancer. The objective of this work is to highlight the importance of measuring indoor concentration of this gas and identify which steps should be taken for achieving radiological protection.

A survey was conducted on the websites of the National Health Surveillance Agency (ANVISA), LAMIN (Mineral Analysis Laboratory), CPRM (Geological Survey of Brazil), Ministry of Health and PubMed. Using the words ‘radon’, ‘lung’, ‘cancer’, and PubMed®, 1,371 results were obtained; when using the words ‘radon’, ‘lung’, ‘cancer’, and with ‘Brazil’ or ‘Brazilians’, only six results were obtained. We emphasise that lung cancer is a major public health problem and the exposure to Rn indoors should be considered as a risk factor for lung cancer in non-smokers. Buildings or houses with high concentrations of Rn should be identified. However, currently in Brazil—a country with great potential for mineral extraction—there are no specific regulated recommendations to control indoor exposure to Rn.

## Introduction

Lung cancer represents the main cause of death from cancer throughout the world. The mean cumulative total survival rate at five years varies between 13% and 21% in developed countries and 7% and 10% in developing countries [1]. The global estimate gave an incidence of 1.82 million new cases of lung cancer for the year 2012. In Brazil, it was responsible for 22,424 deaths in 2011, and 27,330 new cases were expected for the year 2014 [2].

Though its main risk factor continues to be the consumption of tobacco products [3], there has been an increase in the number of cases in non-smoker patients. For this reason, it has become necessary to better understand other risk factors. Among these are passive or second-hand smoking, inflammatory lung disease (such as fibrosis of the lung and chronic obstructive pulmonary disease), exposure to Radon (Rn), asbestos, air pollution and smoke from burning wood [4]. The death rates from lung cancer are declining in developed countries, where tobacco consumption has declined in the last decades. In contrast, the rates of lung cancer and mortality are increasing in developing countries, including many countries of Latin America [1].

Rn has been recognized as the second most common cause of lung cancer in 2005 by the United States Environmental Protection Agency (EPA) [5]. In 2009, the World Health Organization (WHO) in conjunction with EPA launched a first global call-to-action on cancer risk from radon in homes [6]. Thus, it is of fundamental importance to disseminate knowledge about Rn. In Brazil very little information has been released or discussed regarding exposure and prevention of exposure to Rn. This article provides a description of the data found regarding Rn, when and where human contact with the gas occurs, and the policies for prevention that have been implemented in the country.

Rn is an inert natural gas, and is a product of the decay of Radium-226 (Ra-226) [7]. Its concentrations are directly related to the type of geologic formation of a specific region, given that the types of rock that compose these soils can contain smaller or larger quantities of the minerals Uranium (Ur), Thorium (Th), and Radium (Ra), the natural producers of Rn-222 [8].

This gas can escape through fissures or be transported from the subsoil to the surface by means of pipes, holes, and groundwater deposits, and thus become a source of exposure for the people who reside in or frequent these areas [8, 9].

The release of Rn from rock and soil is controlled by mineralogical factors such as solubility and imperfections in the crystalline structure, also specific surface of the minerals that contain uranium. The probability of Rn escaping from the mineral is much greater at the edges of the crystals. The soils generally release more Rn than the rocks, as their component elements are more easily separated [10].

Most exposure to Rn occurs in enclosed environments such as tunnels, caves, mines, houses, and other dwellings. An important form of exposure of individuals inside residences is by inhalation of the Rn isotopes and the products of their decay with a short half-life. It has been proven that high concentrations of Rn within buildings are the result of its release into the atmosphere from the soil through convection [11]. Measures for reducing exposure to Rn include improved ventilation, remediation of Rn, and new construction techniques that reduce the risk of exposure [12, 13]. A variety of Rn mitigation strategies are being used, with different efficacy rates. The ideal strategy depends on the probable source or cause, characteristics of the construction, soil, and climate [13].

In its gaseous state, Rn is colorless, odorless, and tasteless and its particles can be inhaled from the air, and thus penetrate a short distance into the bronchial epithelium, where it can cause biological damage such as mutations to DNA base pairs and chromosomal breakages [13]. Rn is the most important natural source of human exposure to ionizing radiation [9]. Radioactivity due to Rn accounts for 54% of the radiation to which we are exposed daily [14].

Epidemiological studies have demonstrated the synergistic effect between Rn and smoking tobacco [16]. It has been demonstrated that with the same exposure to Rn and its decay products, the risk of lung cancer among smokers is greater than that of non-smokers [17].

## Methods

An extensive survey was conducted between 30 October 2014 and 27 June 2015 using the word ‘radon’ on the websites of the following Brazilian Federal Authorities: National Agency of Health Surveillance (from Portuguese ANVISA), Mineral Analysis Laboratory (from Portuguese LAMIN), Mineral Resources Research Company (from Portuguese CPRM), and Ministry of Health. We have also contacted the ANVISA authorities for further information about national health policies on Rn level control and exposure mitigation.

A PubMed search was also conducted of the principal data published domestically and internationally on the relationship between Rn and lung cancer. A descriptive analysis of the data collected was performed.

## Results and Discussion

When using the words ‘radon’, ‘lung’, ‘cancer’, and PubMed, 1,371 results were obtained; by adding the word ‘Brazil’, only six results were obtained. Of these, two articles dealt with exposure to Rn in coal mines, two were studies conducted outside the country which mentioned Brazil, and the remaining two articles were the only that dealt with studies of Rn in the Brazilian population [18, 19].

Lung cancer in non-smoking patients shows differences in the clinical, pathological, and molecular aspects in comparison to lung cancer in smokers [20]. The principal factors associated with neoplasia of the lung in non-smokers include exposure to known and suspected carcinogenic agents, including Rn, passive smoking, and other air pollutants [21].

The *Environmental Protection Agency* of the United States (EPA) estimates that Rn is the principal cause of lung cancer in non-smokers, and may be responsible for nearly 21,000 deaths from lung cancer per year [14]. A Brazilian study conducted in the city of Poços de Caldas, located in an area with high levels of natural radiation, showed a lifetime increase of 20% in deaths from lung cancer because of exposure to Rn. It was also estimated that 16% of all of the deaths from lung cancer in Poços de Caldas could be attributed to exposure to Rn [18].

Besides EPA and WHO statements, Rn is also classified as a Class I carcinogen by the *International Agency for Research on Cancer* (IARC) [22]. Indoor Rn exposure seems to be a risk factor for all histological types of lung cancer. In individuals diagnosed at a younger age, a higher concentration of residential Rn was identified [23]. According to the *International Commission on Radiological Protection* (ICRP), the management of exposure to Rn is principally based on the application of the principle of optimization below an appropriate reference level of natural radiation (NR). The ICRP recommends 10 millisieverts (mSv) per year as an acceptable dose of radiation absorbed through exposure to Rn. The highest recommended reference level for radiation activity in residences is 300 Bequerels per cubic meter (Bq/m^3^—as an average yearly concentration). The same level is recommended for mixed-use buildings. A specific gradual approach is recommended in work environments: 1) application of the same NR as for dwellings (as long as the corresponding dose is less than 10 mSv/year, mainly because of exposure time); 2) application of the NR of 10 mSv/year, taking the actual conditions of exposure into account; 3) application of the relevant requirements for occupational exposure when, despite the use of possible measures, exposure remains above 10 mSv/year (quantitative criteria), or when the work activity is on a list of occupations with a risk of exposure to Rn (qualitative criteria) [22]. While the action level in the USA is 148 Bq/m^3^ and in Europe it is 200 Bq/m^3^ for new homes, WHO recently proposed a reduction in the exposure levels to below 100 Bq/m^3^ [6].

Three analyses comparing data on individual exposure, including 13 European, 7 North American, and 2 Chinese studies, showed that Rn is a carcinogenic agent in the general population in concentrations found in typical homes, and that there is a linear relationship between the dose and response curves with no evidence of a plateau, as well as evidence of an increased risk even with concentrations below 200 Bq/m^3^ [24, 25]. In contrast, a meta-analysis of 60 publications showed a non-linear association of the dose and response between exposure to environmental Rn and the risk of lung cancer. This increased risk is particularly evident when the cumulative exposure to Rn is well above the recommended concentration limit for a long period of time [26].

The ‘radon manual’ is a key product of the ‘International WHO Radon Project’ launched in 2005. The manual focuses on exposure to residential Rn from a public health perspective and provides detailed recommendations for the reduction of health risks arising from this gas and actions to prevent and mitigate exposure to the same. This publication is intended for countries that seek to develop their national programmes or extend such activities, as well as parties interested in control, such as the construction industries and professionals [6]. It is important to note that should there be an intervention at a location, then a considerable reduction in the concentration of Rn is required, not only reduction but what is needed here is to achieve a level of exposure which is below the lowest limit of the recommended range.

In Brazil, according to the National Agency of Health Surveillance (ANVISA) regulations and legislation currently only cover workers who are directly exposed to large concentrations of the gas such as those in mining, for example. Thus, information on Rn concentrations within buildings, offices, houses and construction materials are not available (personal e-mail received on 19 December 2014).

Marques *et al* (2006) identified a relatively high concentration of Rn in the water, residences, soil, and caves in a Brazilian region, which in some cases reached levels above the maximum limits recommended internationally. The authors suggest that intervention activities be implemented for the dissipation of Rn both in residences and during the collection of water for human consumption [8].

A study conducted in Canada demonstrated that a public health strategy based on testing and mitigation could theoretically prevent 11% of deaths from lung cancer attributable to Rn each year, if in all of the homes tested with levels above the current Canadian standard of 200 Bq/m^3^ were reduced to minimum levels. If the WHO standard of 100 Bq/m^3^ were used, 28% of the deaths from lung cancer attributable to Rn could be avoided annually [23]. A Swedish study showed that nearly 25–30% of the cases of lung cancer attributable to exposure to Rn could be avoided if all of the residential concentrations of Rn above 200 Bq/m^3^ were reduced to 140 Bq/m^3^; while if all of the exposures above 200 Bq/m^3^ were reduced to 100 Bq/m^3^, nearly 35–40% of these cases could be avoided [12]. Hunter *et al* (2015) demonstrated that the mitigation of Rn can have a substantial impact on the risk of lung cancer, even for persons in their sixth decade of life and beyond; in smokers, former smokers, and people who have never smoked, reducing by more than a third the risk of lung cancer induced by Rn [27].

Another study conducted in the American state of Minnesota to assess the effectiveness of soil ventilation systems for 140 randomly selected clients of six professional mitigators found an average concentration of Rn of 280 Bq (10.3 pCi) before mitigation. The average Rn following mitigation in the homes was of 30 Bq/m^3^, with an average reduction of more than 90%. Even years after the mitigation, 97% of these homes had concentrations below the action level of 150 Bq/m^3^ for the United States Environmental Protection Agency [28].

Under Brazilian national regulations, subsection 6.1.3.2 of Regulation CNEN-NN3.01 establishes that ‘in situations of chronic exposure, when relevant action levels have been exceeded, as calculated based on the intervention levels established or approved by the CNEN (National Nuclear Energy Commission), mediatory action must be taken’. An annual dose of 10 mSv must be used as a general reference value for an intervention action in situations of chronic exposure. The CNEN considers intervention to be justified whenever the existing dose is greater than 50 mSv per year [29].

The United States Office of the Surgeon General and EPA recommend that tests be performed in all homes to detect the presence of Rn [30].

As for policies for the prevention of lung cancer, an American study which reviewed 65 control programmes showed that 27 (42%) included terminology specific to Rn, and mentioned the need to improve understanding of Rn as a risk factor. Also included were tests for Rn in homes (n = 21), remediation activities (n = 11), support for Rn policies (n = 13), and assessment of policies (n = 1) [30]. As is the trend in Europe and North America, interior Rn exposure is increasingly recognized in Brazil as an agent that contributes to the risk of lung cancer. Scattered regional surveys conducted in Brazil have shown that the Rn problem may exist in certain areas, but little is known about its possibilities in full extent [31].

## Conclusions

Based on all of the data examined, our conclusion is that Rn must be considered as a risk factor for the development of lung cancer, and major measures must be taken in order to reduce this risk. We recommend that the Ministry of Health in Brazil, along with the responsible agencies, initiate policies for the investigation and mapping of locations in terms of the risk of exposure from this gas. The identification of the areas with high concentrations of exposure to Rn is considered to be of fundamental importance. Directives for the reduction of risk of exposure to this gas, as well as incentives for the construction of buildings to resist the gas and improvements to their ventilation systems are important measures. It is also necessary to initiate policies for increasing awareness and educating the population about what Rn is, its risks, and how the population can avoid it.

## List of abbreviations

ANVISA: National Health Agency of Surveillance (Brazil)
CNEN: National Nuclear Energy Commission (Brazil)
EPA: Environmental Protection Agency (USA)
IARC: International Agency for Research on Cancer
ICRP: International Commission on Radiological Protection
LAMIN: Mineral Analysis Laboratory (Brazil)
WHO: World Health Organisation
Rn: Radon

## Conflicts of Interest

This study does not present any personal or economic conflicts of interest.
